# Validation of administrative data sources for endoscopy utilization in colorectal cancer diagnosis

**DOI:** 10.1186/1472-6963-12-358

**Published:** 2012-10-13

**Authors:** Xue Li, Robert Hilsden, Shakhawat Hossain, John Fleming, Marcy Winget

**Affiliations:** 1 Division of Community Oncology, Cancer Care, Alberta Health Services, Edmonton, Alberta, Canada; 2 Department of Community Health Sciences and Department of Medicine, University of Calgary, Calgary, Alberta, Canada; 3 Department of Public Health Sciences, School of Public Health, University of Alberta, 1500 Sun Life Place, 10123 99 Street NW, Edmonton, Alberta, T5J 3H1, Canada; 4 Present address: Department of Mathematics and Statistics, University of Winnipeg, Winnipeg, Canada

**Keywords:** Colorectal endoscopy, Data validation, Administrative data, Agreement

## Abstract

**Background:**

Validation of administrative data is important to assess potential sources of bias in outcome evaluation and to prevent dissemination of misleading or inaccurate information. The purpose of the study was to determine the completeness and accuracy of endoscopy data in several administrative data sources in the year prior to colorectal cancer diagnosis as part of a larger project focused on evaluating the quality of pre-diagnostic care.

**Methods:**

Primary and secondary data sources for endoscopy were collected from the Alberta Cancer Registry, cancer medical charts and three different administrative data sources. 1672 randomly sampled patients diagnosed with invasive colorectal cancer in years 2000–2005 in Alberta, Canada were included. A retrospective validation study of administrative data for endoscopy in the year prior to colorectal cancer diagnosis was conducted. A gold standard dataset was created by combining all the datasets. Number and percent identified, agreement and percent unique to a given data source were calculated and compared across each dataset and to the gold standard with respect to identifying all patients who underwent endoscopy and all endoscopies received by those patients.

**Results:**

The combined administrative data and physician billing data identified as high or higher percentage of patients who had one or more endoscopy (84% and 78%, respectively) and total endoscopy procedures (89% and 81%, respectively) than the chart review (78% for both).

**Conclusions:**

Endoscopy data has a high level of completeness and accuracy in physician billing data alone. Combined with hospital in/outpatient data it is more complete than chart review alone.

## Background

Databases that are developed and maintained for administrative purposes are frequently used in population health research and disease surveillance because of their availability, generality, cost-effectiveness and large population encompassed. The quality of administrative data, however, is often questioned when the data are employed in health outcomes research or quality measurement [1-9]. It is, therefore, important to validate administrative data in order to assess potential sources of bias in outcome evaluation and to prevent dissemination of misleading or inaccurate information [10].

Validation studies of administrative data have primarily focused on diagnosis of disease [10-20]. In cancer research, however, the primary data source used for identifying cancer cases is typically a well-established cancer registry; administrative data are not usually used or needed to identify cancer cases. Administrative data, however, can be very valuable in identifying key procedures received during a cancer patient’s care trajectory in order to evaluate the care received [21,22], to understand patterns of service delivery [23], and/or to predict future resource needs [24]. Validating the potential administrative data sources to be used in such studies should be a critical component of the study itself.

The purpose of the study was to validate the completeness and accuracy of endoscopy data in several administrative data sources in the year prior to colorectal cancer diagnosis as part of a larger project focused on evaluating the quality of the pre-diagnostic care trajectory of colorectal cancer patients with respect to tests received and timing of them.

## Methods

### Inclusion criteria

An approximate 20% random sample of all residents of Alberta, Canada diagnosed with invasive colon cancer (International Classification of Diseases for Oncology (ICD-O) [25] codes: c18, excluding appendix) or rectal cancer (ICD-O c19 and c20) in years 2000 to 2005, stratified by stage and year of diagnosis, were identified from the Alberta Cancer Registry and included in the study. Patients were excluded for the following reasons: stage 0 cancer; histology that are not staged according to the Collaborative Staging Guidelines [26]; or missing the unique lifetime identifier (ULI). The ULI is a unique number assigned to all members of the Alberta Health Care Insurance Program (AHCIP), the publicly-funded provincial healthcare insurance plan in Alberta. The ULI is, therefore, used as the anonymized patient identifier in all provincial administrative databases in Alberta and was used to link data across data sources for the study.

### Chart review data

A chart review using the cancer clinic medical chart was conducted to identify dates of endoscopy prior to and including the date of diagnosis. Cancer medical charts are initially created for all patients diagnosed with cancer by the Alberta Cancer Registry for use in coding cases. They include procedure reports such as those for pathology, surgery, or endoscopy, plus referral letters and dictation notes, if the patient is seen by an oncologist; thus a cancer chart exists for every patient diagnosed with cancer in the province. The following data were abstracted from the charts: date and type of endoscopy; result (cancer, suspicious, not cancer); and source of information (letter, dictation notes, report).

### Administrative health databases

Endoscopy data were obtained from three provincial administrative databases, the first two of which conform to national reporting standards: 1) the Discharge Abstract Database (hospital inpatient data) which records information on all admissions to hospitals in Alberta; 2) the Ambulatory Care Classification System Database (hospital outpatient data), which contains information on all outpatient visits that occurred in hospitals, such as visits to hospital-based physicians’ offices, hospital endoscopy units, and emergency departments; and 3) the Physician Billing database, which contains all billing claims submitted by physicians remunerated on a fee-for-service basis and “shadow” billing submitted by physicians employed through the Alternate Relationship Plan (ARP). The latter group of physicians comprises a small number of physicians in one city during the time period of this study. From each data source, dates and codes for endoscopy procedures were identified that occurred within one year prior to colorectal cancer diagnosis for each patient included in the study. The timeframe of one year prior to diagnosis was determined based on a sensitivity analysis we conducted comparing endoscopies found 12, 18, or 24 months prior to colorectal cancer diagnosis; roughly the same number were found regardless of the time frame, therefore we used one year as the cutoff.

Each data source uses a different coding system and coding systems changed from ICD-9 to ICD-10 in April 2002 for the hospital datasets. In order to identify endoscopy codes from each data source appropriately, a literature review was conducted and input from local physicians was obtained. Since our purpose was to identify all lower gastrointestinal endoscopies regardless of purpose, all codes that indicated use of an endoscope were included. The endoscopy procedure codes included in the study from each data source are listed in Additional file 1.

### Combined administrative dataset

The three administrative datasets were combined using the assumption that if an endoscopy was identified in any source then it was assumed to have occurred. This is because: 1) we expect that most patients will have had an endoscopy prior to colorectal cancer diagnosis and 2) it is unlikely that an endoscopy would be identified in any of the data sources if it was not actually performed; that is, the probability of a false positive is low. The data were combined in such a way as to minimize error in identifying unique endoscopies and also to assess accuracy with respect to the date of the endoscopy in the various data sources. In practice, it would be reasonable for an endoscopy code for the same event to appear in a hospital inpatient record and physician billing record or hospital outpatient record and physician billing record. Coding rules and practices should prevent the same event from being coded in both hospital inpatient and outpatient data unless an error is made. This is because procedures that happen to patients as outpatients should not be entered as a procedure as an inpatient (and vice versa), even if the patient is admitted the same day. Similarly, it is unlikely that a patient would undergo more than one endoscopy on the same day. Furthermore, dates for events in the hospital databases are expected to be accurate because the data are entered and coded by trained health records technicians. Physician billing, however, is more prone to error with respect to both the accuracy of the code and the date. In order to minimize the chance of counting a given endoscopy more than once and minimize the chance of counting two or more events as one when combining the datasets, the following rules were applied: 1) if an endoscopy appeared in both the inpatient and outpatient datasets for the same individual and date it was considered to be the same endoscopy; 2) if an endoscopy in the physician billing data was within three days of an endoscopy in either hospital dataset then it was counted as the same endoscopy. These rules were tested against rules using three and seven day windows, respectively, with the result that there was minimal difference in the number of unique endoscopies identified. If a patient did not appear in a dataset then the patient was assigned to the “No Endoscopy” category for that particular dataset.

### Gold standard

The gold standard dataset was created by combining all administrative datasets and the chart review data. If a procedure was identified in any data set, it was considered to have occurred in the gold standard. The cancer clinic medical chart was not adopted as the gold standard because, even though information that is collected by the cancer registry to code and stage patients is in these charts, it is possible that an endoscopy that did not result in removal of tissue would be missed. Furthermore, although pathology reports are obtained when possible, some information may be obtained from referral letters or dictation notes which are subject to error. For this reason a gold standard was created to maximize the probability of identifying all unique endoscopies conducted in the year prior to colorectal cancer diagnosis. The same rules and assumptions that were followed to create the combined administrative dataset were applied in creating the gold standard: 1) if an endoscopy appeared in either data source then it was assumed to have occurred (probability of false-positive is low) and 2) endoscopies in the chart review dataset that were within three days of the date of an endoscopy in the combined administrative dataset were counted as the same endoscopy.

### Data analysis

The measures to evaluate the completeness of the data were calculated at two levels: 1) comparing the total number of patients that underwent endoscopy and 2) comparing the total number of endoscopy procedures identified in each data source. The following descriptive statistics were calculated regarding patients who received an endoscopy and endoscopies identified from each dataset using the respective totals identified in the gold standard as the denominators for percentages: 1) total number and percent, 2) the number and percent identified from one and only one data source, by data source and, 3) the number and percent identified from one and only one of the administrative data sources, by administrative data source; note, these may have also been identified from the chart review. The purpose of this latter set of statistics is to indicate the extent to which each administrative data source contributes uniquely in the absence of a chart review. The percentage of endoscopy procedures that had exact date matches was used to determine the accuracy of the data.

In order to assess the likelihood that endoscopies were missed, clinical characteristics and health care service utilization were compared between patients who had an endoscopy to those who did not. Specifically, patient age at diagnosis, disease stage, type of first colorectal cancer-related healthcare visit (pre-diagnostic or not), and time from diagnosis to death were explored. These were selected because they were considered to be potentially relevant reasons individuals may not receive an endoscopy prior to colorectal cancer diagnosis. Statistical significance was defined at the α=0.05 level. All analyses were performed using statistical software SAS 9.1.3 (SAS Institute, Cary, NC, USA) or STATA/SE 10.0 (StataCorp LP, TX, USA).

## Results

There were 1672 patients diagnosed with colorectal cancer in years 2000–2005 who were randomly selected and included in the study. Table 1 compares the patient characteristics and health service utilization in the entire population of colorectal cancer patients diagnosed in Alberta in years 2000–2005 versus the sample of 1672 patients. The sample of patients included in the study is representative of the population on the factors examined.

**Table 1:** Patient characteristics of cohort and sample

**Characteristics**	**Cohort (%)**	**Sample (%)**
**Total Number**	8310 (100)	1672 (100)
**Age at diagnosis**		
Mean [Median]	69 [70]	69 [70]
<=65	3042 (37)	643 (38)
66-74	2172 (26)	437 (26)
>=75	3096 (37)	592 (35)
**Stage at diagnosis**		
I-III	5500 (66)	1145 (68)
IV or Unknown	2810 (34)	527(32)
**Type of 1**^**st**^**colorectal cancer-related visit**^1^		
Late^2^ event	2545 (31)	506 (30)
(Pre)Diagnostic event	5765 (69)	1166 (70)
**Time from diagnosis to death (days)**^3^		
Mean [Median]	661 [453]	711 [466]
<= 1 month	491 (11)	84 (10)
1 - 3 months	472 (11)	90(10)
>= 3 months	3431(78)	710 (80)

^1^ Limited to healthcare visits within 1 year prior to diagnosis of colorectal cancer.

^2^ Late event is defined as a visit that included treatment-related events (e.g., surgery, palliative care) rather than diagnostic or pre-diagnostic events (e.g., radiology, symptoms). Patients who did not have any colorectal cancer-related healthcare visits are also included in this group.

^3^ Includes only patients who died on or before March 31, 2009.

Table 2 describes the endoscopy data obtained from the chart review. There were 1506 endoscopies identified from the patient charts. Over half (65%) of the data were abstracted from pathology reports, nearly 30% of the endoscopies were sigmoidoscopies, and the results for 93% of the endoscopies were a cancer diagnosis.

**Table 2:** Summary of endoscopy information from the chart review

**Description**	**N (%)**
**# of Endoscopies**	1506 (100)
**Type of Endoscopy**	
Colonoscopy	1068 (71)
Sigmoidoscopy	438 (29)
**Results**	
Cancer	1406 (93)
Not Cancer	55 (4)
Suspicious	45 (3)
**Source of Information**	
Pathology Report	979 (65)
Letter or Dictation Note	527 (35)

Table 3 summarizes the total number of patients and endoscopy procedures identified from each data source relative to the gold standard and the number and percent that were uniquely identified from each data source. Out of 1672 patients included in the study, a total of 1937 endoscopy procedures conducted on 1443 patients (86%) were identified by the gold standard. The combined administrative data identified 1732 (89%) of the endoscopy procedures and 1403 (84%) of the patients, this was somewhat higher than the endoscopies (1506, 78%) and patients who had an endoscopy (1310, 78%) identified by chart review alone. The physician billing was the best single administrative data source with similar completeness to the chart review alone identifying 1566 (81%) of endoscopies conducted and 1300 (78%) of the patients.

**Table 3:** Total number of patients and endoscopies identified by different data sources

**Data Source**	**All Patients**	**Patients with Endoscopy**	**Patients without Endoscopy**	**Total Number of Endoscopies**
	**n (%)**	**n (%)**^1^	**n (%)**^1^	**n (%)**^2^
	1672 (100)			
**Gold Standard**		1443 (86)	229 (14)	1937 (100)
**Chart Review**		1310 (78)	362 (22)	1506 (78)
Unique^3^		40 (3)		205 (11)
**Physician Billing**		1300 (78)	372 (22)	1566 (81)
Unique^3^		24 (2)		152 (8)
Unique admin data^4^		91 (7)		125 (6)
**Hospital Inpatient**		326 (19)	1346 (81)	354 (18)
Unique^3^		9 (<1)		33 (2)
Unique admin data^4^		56 (4)		39 (2)
**Hospital Outpatient**		1021 (61)	651 (39)	1126 (58)
Unique^3^		4 (<1)		30 (2)
Unique admin data^4^		32 (2)		63 (3)
**Combined Administrative**		1403 (84)	269 (16)	1732 (89)
Unique^3^		133 (9)		431 (22)

^1^ Percent based on all patients included in the study (n=1443).

^2^ Percent based on total number of endoscopies identified in gold standard (n=1937).

^3^ Number (percentage) identified in one and only one data source.

^4^ Number (percentage) identified in one and only one of the administrative data sources, may also be identified from the chart review.

Similar to the results of the overall completeness of the single data sources, the chart review identified the most patients (40) and endoscopies (205) uniquely and the physician billing identified the most of the individual administrative data sources: 91 patients and 125 endoscopies. The combined administrative data, however, identified 133 patients (9%) and 431 endoscopies (22%) that were not found in the chart review.

Patients identified in the hospital inpatient data tended to be older and have higher stage than those identified in the other data sources: 25% of patients with an endoscopy in the inpatient data were 80 years of age or older compared to 15-20% in the other single data sources and 33% had stage IV disease compared to 16-19% in the other single data sources.

Of the 1732 endoscopies identified in the combined administrative dataset, 1289 (74%) were found in the physician billing plus at least one of the hospital datasets and 1254 (97%) of these had an exact match for the date of the procedure (not shown in the tables), illustrating near-perfect agreement between the physician billing and hospital data with respect to dates of endoscopy procedures.

Table 4 describes the level of agreement between data sources with respect to number of patients who had an endoscopy procedure and number of endoscopies identified. The highest level of agreement was between the chart review and combined administrative data with 90% agreement on patients identified (or not) with endoscopy and 71% agreement on endoscopies identified (or not). Agreement between physician billing and chart review was only slightly less at 85% for the patient level and 69% at the endoscopy level. The lowest agreement was between the hospital inpatient and outpatient data which was 26% at the patient level and 34% at the endoscopy level. Most of the agreement at both the patient and endoscopy levels between these two data sources was due to the “no” cells, that is, 384 of the 443 patients (87%) for which there was agreement did not have an endoscopy in either data source. Agreement between the physician billing and hospital inpatient was only slightly better at 37% for both patient and endoscopy level, however, the agreement was roughly equally split due to consistency in identifying patients who had (283 patients) or did not have (329 patients) an endoscopy.

**Table 4:** Number and percent agreement of patients and endoscopies across data sources

	**Physician Billing**	**Hospital Inpatient**	**Hospital Outpatient**	**Combined Administrative**
	**n (%)**	**n (%)**	**n (%)**	**n (%)**
**Chart Review**				
Patient	1422 (85)	618 (37)	1225 (73)	1499 (90)
Endoscopy	1790 (69)	860 (40)	1382 (64)	1530 (71)
**Physician Billing**				
Patient		612 (37)	1269 (76)	
Endoscopy		808 (37)	1538 (71)	
**Hospital Inpatient**				
Patient			443(26)	
Endoscopy			736 (34)	

In order to assess the likelihood that endoscopies were missed, even in the Gold Standard, clinical characteristics and health care service utilization were compared between patients who had an endoscopy (n=1442) to those who did not (n=230) according to the Gold Standard. Results are shown in Table 5. Patients who did not have a record of endoscopy were more likely to be diagnosed with stage IV disease (P <0.0001), had shorter survival from diagnosis (P <0.0001), and were more likely for their first colorectal-related health care visit in the year prior to their diagnosis to be a “late” event (P <0.0001) than those who had an endoscopy record. “Late” events were defined as visits that involved only services expected after cancer diagnosis has been made, such as surgery or palliative care, and did not include any expected pre-diagnostic services such as endoscopy, radiology, or presentation with symptoms.

**Table 5:** Patient characteristics of those who had an endoscopy prior to colorectal cancer diagnosis compared to those who did not in the Gold Standard dataset

**Characteristics**	**Did not have endoscopy n (%)**	**Had endoscopy n (%)**	***P*value**
**Total Number**	229 (100)	1443 (100)	
**Age at diagnosis**			***0.24***
Mean [Median]	70 [72]	68 [69]	
<=65	84 (37)	559 (39)	
66-74	53 (23)	384 (26)	
>=75	92 (40)	500 (35)	
**Stage at diagnosis**			***<.0001***
I-III	110 (48)	1035 (72)	
IV or Unknown	119 (52)	408 (28)	
**Type of 1**^**st**^**colorectal cancer-related visit**^1^			***<.0001***
Late^2^ event	104 (45)	402 (28)	
(Pre)Diagnostic event	125 (55)	1041 (72)	
**Time from diagnosis to death (days)**^3^			***<.0001***
Mean [Median]	467 [200]	771 [546]	
<= 1 month	41 (24)	43 (6)	
1 - 3 months	20 (11)	70 (10)	
>= 3 months	111 (64)	599 (84)	

^1^ Limited to healthcare visits within 1 year prior to diagnosis of colorectal cancer.

^2^ Late event is defined as a visit that included treatment-related events (e.g., surgery, palliative care) rather than diagnostic or pre-diagnostic events (e.g., radiology, symptoms). Patients who did not have any colorectal cancer-related healthcare visits are also included in this group.

^3^ Includes only patients who died on or before March 31, 2009.

## Discussion

The purpose of this study was to determine the completeness and accuracy (with respect to dates) of various administrative data sources in identifying endoscopies in the year prior to colorectal cancer diagnosis. The findings of the study support the use of physician billing alone or combined with hospital inpatient and outpatient data as reasonable data sources for identifying patients who have had at least one endoscopy in the year prior to colorectal cancer diagnosis but a combination of hospital and physician billing data is recommended to identify the total number of endoscopies received. This conclusion is restricted to the setting in which the majority of physicians performing endoscopy are remunerated on a fee-for-service basis in the single-payer health care system and/or in which salaried physicians submit claims for procedures performed. Hospital data alone are not good sources for this information because a significant number and percentage of endoscopies occur outside the hospital.

Physician billing data are created for the purpose of remunerating physicians who are paid on a fee-for-service schedule. The completeness of the data is likely to be high if specific fee code exists for a well-defined procedure (such as endoscopy) and physicians have the incentive to record the procedure accurately in their claim for their fee reimbursement. Accuracy of the physician billing data, therefore, is subject to the fee code policy. The results of studies based on physician billing data could easily be misinterpreted if certain procedure codes are unknowingly under or over claimed due to variances in reimbursement for related and/or similar procedures. Caution is, therefore, needed in the conduct and interpretation of studies based on physician billing data; strong understanding of the way in which physicians use billing codes and the percentage of physicians who perform the procedure of interest that bill for it is needed. Validation of the data is also critical.

One of the shortcomings to our method of validation was the lack of independence between our gold standard dataset and our comparison data sets. Our study did not evaluate the accuracy of the endoscopy with respect to type of exam (colonoscopy vs. sigmoidoscopy) or reason for exam (screening vs. diagnosis), however, a few studies have done so. Not surprisingly, they have all found that administrative data are not adequate for assessing this level of specificity with respect to type or reason for exam [9,27-29]. For instance, Schenck et al. found Medicare claims to be accurate for identifying endoscopies but not for distinguishing screening from diagnostic tests. This is at least in part due to the absence of billing codes that are specific to screening tests but even if implemented, the fee code would need to be comparable to the diagnostic fee code in order to provide physicians incentive to use it.

As mentioned, it is expected that patients with colorectal cancer would have at least one endoscopy procedure prior to their diagnosis as endoscopy is the most common definitive diagnostic procedure. Fourteen percent of the patients in the study, however, did not have any endoscopy record in the gold standard. To examine the likelihood that endoscopies were missed, even in the gold standard, we explored clinical characteristics and other health service utilization of patients who did not have an endoscopy procedure identified in any of the data sources (n=230). About half of these patients presented with stage IV disease and about half had at least one colorectal-related symptom at a healthcare visit within one year prior to their colorectal cancer diagnosis. One would expect that patients who had colorectal-related symptoms prior to diagnosis would have had an endoscopy so it is possible that endoscopies for these patients (n=110) were incorrectly missed. Alternatively, it is possible that some of these patients were diagnosed via an alternative route such as by a CT scan that identified metastatic disease or as an emergency patient that went straight to surgery. The high percentage of patients with stage IV disease who did not have an endoscopy recorded makes these alternative diagnostic routes likely. Additionally, some patients may have had an endoscopy in another province. A minority of cancer patients receives treatment outside the province and some may receive some or all of their diagnostic work-up outside the province as well. Given these possible scenarios, it is likely that at least 100 to 125 patients (5–7.5%) of the total patient cohort did not have an endoscopy at all or in Alberta prior to their colorectal cancer diagnosis. The patients who received endoscopies within Alberta as part of the process in diagnosing colorectal cancer, therefore, seem to have been properly identified in the gold standard created for the study combining chart review and administrative data sources.

## Conclusion

Usually the gold standard for validation of administrative data is a disease registry database or medical records [10,12,18,22,30,31]. We chose to combine the information from chart review and each administrative dataset because of recognized limitations to each data source on its own for identifying endoscopies and potential inaccuracies of dates. Additionally, because it is expected that all but a small minority of patients diagnosed with colorectal cancer would have at least one endoscopy in the year prior to diagnosis we were confident that the probability of a false positive in any data source would be negligible. The findings of this study with respect to completeness and accuracy of data sources should be generalizable across Canada and in other jurisdictions in which endoscopies are reimbursed via fee-for-service and similar datasets exist. This is because in Canada, the inpatient and outpatient databases are standardized nationally, even though they are prepared provincially, and have ongoing quality assessments made to them nationally [32]. Furthermore, we expect the methodology for creating a gold standard to be appropriate in similar scenarios in which the procedure is well-defined, is expected to occur in the majority of the population, and for which a true gold standard does not exist. In the absence of an official registry database for endoscopy procedures, physician billing combined with hospital data is the most complete source of information to identify endoscopies.

## Abbreviations

AHCIP: Alberta Health Care Insurance Program; ARP: Alternate Relationship Plan; ICD-O: International Classification of Diseases for Oncology; ULI: Unique lifetime identifier.

## Competing interests

The authors declare that they have no competing interests.

## Authors’ contributions

XL and SH contributed to the statistical analysis, interpretation of data and drafting of the manuscript. RH helped obtain funding, contributed to the study design; interpretation of data; critical revision of the manuscript for important intellectual content; and provided clinical input. JF contributed to the statistical analysis. MW was the overall supervisor of the study and as such contributed in all aspects of the study including obtaining funding, overseeing the analysis, interpretation, critical revision and final approval of the manuscript. All authors read and approved the final manuscript.

## Pre-publication history

The pre-publication history for this paper can be accessed here:

http://www.biomedcentral.com/1472-6963/12/358/prepub

## Supplementary Material

Additional file 1 Colorectal endoscopy procedure codes. Click here for file
