# Supplementary material for: Validation of administrative data sources for endoscopy utilization in colorectal cancer diagnosis
Source: BMC Health Serv Res. 2012 Oct 13;12:358. doi: 10.1186/1472-6963-12-358 (PMC3508878; doi:10.1186/1472-6963-12-358)
Supplement: Additional file 1 — Colorectal endoscopy procedure codes. [file 1472-6963-12-358-S1.doc]

**Appendix A: Colorectal Endoscopy Procedure Codes**

| **The Canadian Classification of Procedures (CCP) – physician billing, all years:** | |
| --- | --- |
| 01.22 | Other nonoperative colonoscopy {Other nonoperative colonoscopy, diagnostic only} |
| 01.24A | Other nonoperative proctosigmoidoscopy {Rigid proctosigmoidoscopy} |
| 01.24B | Other non-operative proctosigmoidoscopy {Flexible proctosigmoidoscopy} |
| 57.21A | Fulguration of lesion of large intestine {Polypectomy of large intestine, additional benefit} |
| 60.21A | Fulguration of rectal lesion or tissue (with cautery) {Fulguration of rectal carcinoma} |
| 60.82A | Other biopsy of rectum {Rectal biopsy with rigid sigmoidoscope} |
| 60.82B | Other biopsy of rectum {Rectal biopsy with flexible sigmoidoscope} |
|  | |
| **International Statistical Classification of Diseases, Injuries, and Causes of Death, Ninth Revision -Clinical Modification(ICD-9-CM) – hospital data 1994 to 31 March 2002** | |
| 4521 | Transabd endoscopy large intest |
| 4522 | Endosc large intest artif stoma |
| 4523 | Colonoscopy |
| 4524 | Flexible Sigmoidoscopy |
| 4525 | Closed biopsy large intestine |
| 4542 | Endosc polypectomy large intest |
| 4543 | Endosc destr oth les lrg intest |
| 4823 | Rigid proctosigmoidoscopy |
| 4824 | Closed Biopsy of Rectum |
| 4836 | (Endo) polypectomy of rectum |
|  |  |
| **The Canadian Classification of Health Interventions (CCI) – hospital data, 1 April 2002 to present:** | |
| 1NM59BAAG | Destruction, large intestine using endoscopic per orifice approach and laser |
| 1NM59BAGX | Destruction, large intestine using endoscopic per orifice approach and device NEC [e.g. electrocautery] |
| 1NM59BAHB | Destruction, large intestine using endoscopic per orifice approach and heat probe |
| 1NM87BA | Excision partial, large intestine endoscopic per orifice approach Simple excisional technique |
| 1NQ59BAAD | Destruction, rectum endoscopic per orifice approach using cryoprobe |
| 1NQ59BAGX | Destruction, rectum endoscopic per orifice approach using device NEC |
| 1NQ59BAX7 | Destruction, rectum using endoscopic per orifice approach and chemical cautery agent |
| 1NQ87BA | Excision partial, rectum endoscopic per orifice approach closure by apposition technique [e.g. suturing, stapling] or no closure required (for tissue regeneration) |
| 2NM70BA | Inspection, large intestine using endoscopic per orifice approach (or via stoma) |
| 2NM70BN | Inspection, large intestine using endoscopic per orifice approach and laser assisted optical "biopsy" |
| 2NM71BA | Biopsy, large intestine using endoscopic per orifice approach (or via stoma) |
| 2NM71BR | Biopsy, large intestine using endoscopic per orifice brush biopsy or washing |
| 2NQ70BA | Inspection, rectum using endoscopic per orifice approach |
| 2NQ71BA | Biopsy, rectum using endoscopic per orifice approach |
| 2NQ71BG | Biopsy, rectum using endoscopic per orifice rectal suction |
| 2NQ71BR | Biopsy, rectum using endoscopic per orifice with brush biopsy or washing |
| 2NQ71CA | Biopsy, rectum per orifice approach NOS |
| 2OW70BA | Inspection, surgically constructed sites in digestive & biliary tract using endoscopic per orifice (or via stoma) approach |
